# Elastin-derived scaffolding associated or not with bone morphogenetic protein (BMP) or hydroxyapatite (HA) in the repair process of metaphyseal bone defects

**DOI:** 10.1371/journal.pone.0231112

**Published:** 2020-04-20

**Authors:** Marcelo de Azevedo e Sousa Munhoz, Karina Torres Pomini, Ana Maria de Guzzi Plepis, Virginia da Conceição Amaro Martins, Eduardo Gomes Machado, Renato de Moraes, Fernando Bento Cunha, Arnaldo Rodrigues Santos Junior, Guinea Brasil Camargo Cardoso, Marco Antonio Hungaro Duarte, Murilo Priori Alcalde, Daniela Vieira Buchaim, Rogerio Leone Buchaim, Marcelo Rodrigues da Cunha

**Affiliations:** 1 Department of Morphology and Pathology, Medical College of Jundiai, Jundiaí, São Paulo, Brazil; 2 Interunit Postgraduate Program in Bioengineering (EESC/FMRP/IQSC), University of São Paulo (USP), São Carlos, São Paulo, Brazil; 3 Department of Biological Sciences, Bauru School of Dentistry, University of São Paulo (FOB/USP), Bauru, São Paulo, Brazil; 4 São Carlos Institute of Chemistry, University of São Paulo (USP), São Carlos, São Paulo, Brazil; 5 Center of Natural and Human Sciences, Federal University of ABC (UFABC), São Bernardo do Campo, São Paulo, Brazil; 6 Materials Engineering Department, Faculty of Mechanical Engineering, State University of Campinas, Campinas, São Paulo, Brazil; 7 University Center Nossa Senhora do Patrocínio (CEUNSP), Cruzeiro do Sul University (UNICSUL), Itu, São Paulo, Brazil; 8 Department of Dentistry, Endodontics and Dental Materials, Bauru School of Dentistry, University of São Paulo (FOB/USP), Bauru, São Paulo, Brazil; 9 Health Sciences Center, Sacred Heart University Center (UNISAGRADO), Bauru, São Paulo, Brazil; 10 Postgraduate Program in Structural and Functional Interactions in Rehabilitation, University of Marilia (UNIMAR), Marília, São Paulo, Brazil; 11 Medical School, University Center of Adamantina (UniFAI), Adamantina, São Paulo, Brazil; Università degli Studi della Campania, ITALY

## Abstract

Tissue engineering represents a promising alternative for reconstructive surgical procedures especially for the repair of bone defects that do not regenerate spontaneously. The present study aimed to evaluate the effects of the elastin matrix (E24/50 and E96/37) incorporated with hydroxyapatite (HA) or morphogenetic protein (BMP) on the bone repair process in the distal metaphysis of rat femur. The groups were: control group (CG), hydrolyzed elastin matrix at 50°C/24h (E24/50), E24/50 + HA (E24/50/HA), E24/50 + BMP (E24/50/BMP), hydrolyzed elastin matrix at 37°C/96h (E96/37), E96/37 + HA (E96/37/HA), E96/37 + BMP (E96/37/BMP). Macroscopic and radiographic analyses showed longitudinal integrity of the femur in all groups without fractures or bone deformities. Microtomographically, all groups demonstrated partial closure by mineralized tissue except for the E96/37/HA group with hyperdense thin bridge formation interconnecting the edges of the ruptured cortical. Histologically, there was no complete cortical recovery in any group, but partial closure with trabecular bone. In defects filled with biomaterials, no chronic inflammatory response or foreign body type was observed. The mean volume of new bone formed was statistically significant higher in the E96/37/HA and E24/50 groups (71.28 ± 4.26 and 66.40 ± 3.69, respectively) than all the others. In the confocal analysis, it was observed that all groups presented new bone markings formed during the experimental period, being less evident in the CG group. Von Kossa staining revealed intense calcium deposits distributed in all groups. Qualitative analysis of collagen fibers under polarized light showed a predominance of red-orange birefringence in the newly regenerated bone with no difference between groups. It was concluded that the E24/50 and E96/37/HA groups promoted, with greater speed, the bone repair process in the distal metaphysis of rat femur.

## Introduction

In recent decades, the search for therapeutic strategies has been increasing in order to promote the innate regenerative potential of deteriorating tissues and restore morphological and functional integrity [1]. Thus, tissue engineering represents a promising alternative for reconstructive surgical procedures, especially for the repair of bone defects that do not regenerate spontaneously [2].

Several types of materials have been developed as substitutes for autogenous grafts due to their well-established limitations, such as the possibility of donor site morbidity, risk of infection and limited amount of material available [3].

In this context, synthetic bone substitutes have become potential materials for clinical applications because they have good biocompatibility with surrounding tissues and chemical stability in body fluid [4]. Thus, synthetic hydroxyapatite (HA) ceramics are often used in orthopedic and craniofacial repairs because they form a mechanically stable interface and have controlled and interconnected pore size, which favors the conduction of osteoprogenitor cells *in situ* [1].

Natural polymers, including the auricular cartilage-derived elastin matrix, referred to as third-generation biomaterials, have aroused interest in providing specific interactions with cellular integrins and thus direct cell proliferation and differentiation, as well as synthesis and organization of the extracellular matrix [5] In addition, elastin microfibrils, the essential protein structure of the extracellular matrix, are interconnected by crosslinking which gives the material the characteristic of a three-dimensional porous framework [6].

Another approach for *in situ* tissue regeneration involves the use of biomaterials as intelligent delivery systems for biomolecular therapy using drugs, cells or growth factors such as bone morphogenetic protein BMP [7]. Thus, diffusion BMPs induce cell differentiation and stimulate other cells to produce additional growth factors, which in turn stimulate several generations of growing cells [8].

Taken together, these findings suggest that the combined treatment of elastin and hydroxyapatite matrix or BMP may favor the process of bone defect consolidation due to possible synergistic action [9]. However, there are no scientific studies of this association in long bone repair. To this end, the present study aimed to investigate the effects of the elastin matrix incorporated into hydroxyapatite or morphogenetic protein on the bone repair process in the distal metaphysis of rat femur.

## Material and methods

### Materials

#### Elastin matrix derived from bovine auricular cartilage

**Fig 1:**
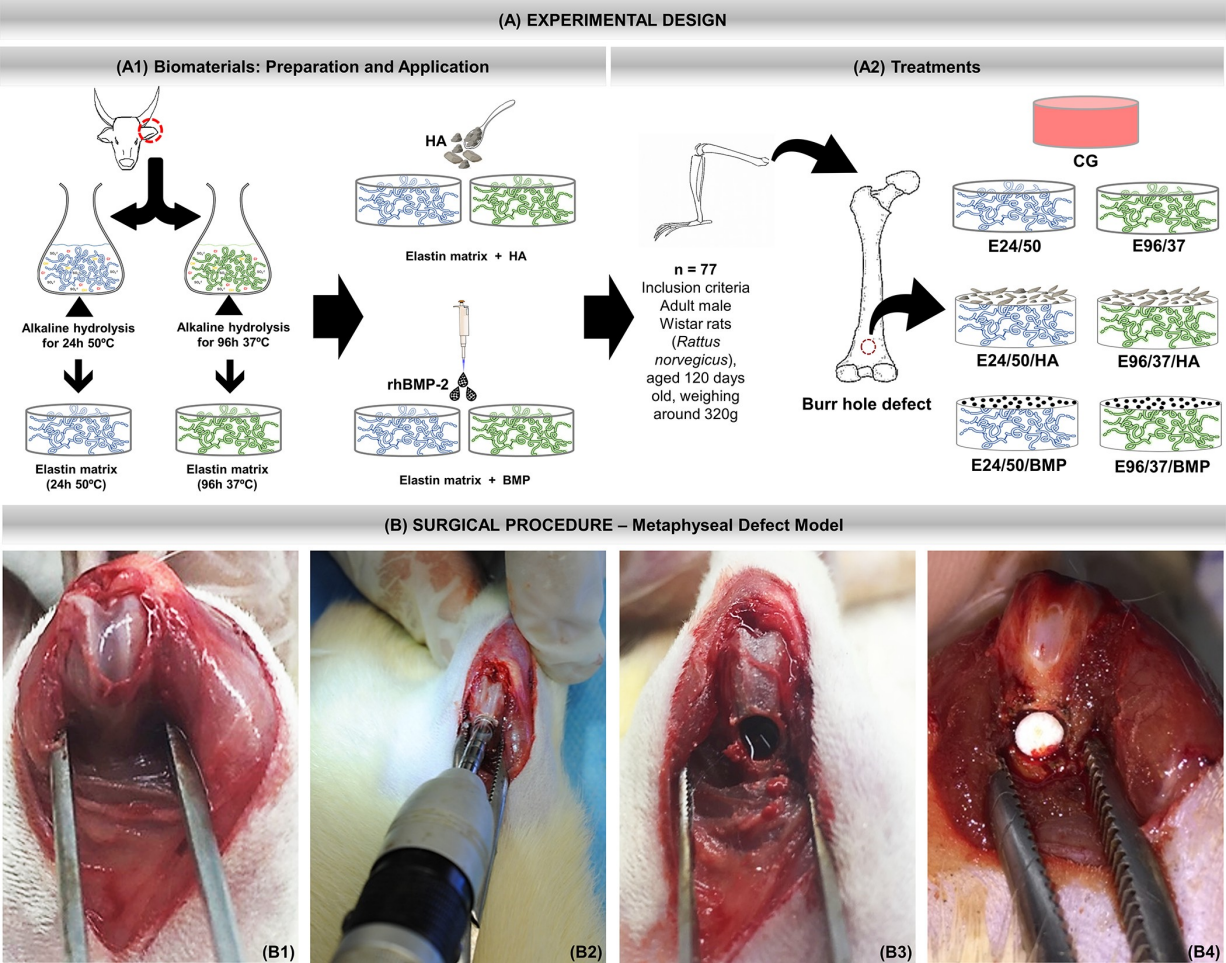
(A) Experimental design. (A1) Preparation and application of biomaterials. Hydrolysis process of bovine atrial cartilage in alkaline solution at 50°C for 24 hours and 96 hours at 37°C. After obtaining the elastin matrices, either synthetic hydroxyapatite (HA) ceramics or human recombinant morphogenetic protein two are incorporated. (A2) Treatments. Inclusion criteria: 77 male rats, *Rattus norvegicus*, Wistar, 120 days old and average weight of 320 grams were randomly separated into 7 groups.: CG—Control group (n = 11)–metaphyseal defect filled with blood clot; E24/50 (n = 11)—hydrolyzed elastin membrane filled metaphyseal defects for 24h at 50° C; E24 /50/HA (n = 11)—hydrolyzed elastin membrane filled metaphyseal defects for 24h at 50°C associated with hydroxyapatite; E24/50/BMPP (n = 11) -metaphyseal defects filled with elastin membrane hydrolyzed for 24h at 50°C associated with BMP; E96/37 (n = 11)—hydrolyzed elastin membrane filled metaphyseal defects for 96h at 37°C; E96/37/HA (n = 11)—Elastin membrane filled metaphyseal defects hydrolyzed for 96h at 37°C associated with hydroxyapatite; E96/37/BMP (n = 11)—metaphyseal defects filled with elastin membrane hydrolyzed for 96h at 37°C associated with BMP. (B) Surgical procedure—Metaphyseal Defect Model. B1) Medial parapatellar incision in the right femur followed by arthrotomy, lateral patellar dislocation and visualization of the femoral condyles. (B2) Metaphyseal Defect Model made with 3 mm diameter trephine drill. (B3) Circular osteotomy in the anterior metaphyseal region of the distal femur. (B4) Defect filled with elastin matrix.

The elastin matrices used were prepared by the Biochemistry and Biomaterials Group of the São Carlos Institute of Chemistry—USP, by adapting the methodology used to obtain collagen [10]. Briefly, the cartilages were treated with an alkaline solution (hydrolysis) containing salts (sulfates and chlorides) and alkaline earth alkali metal hydroxides at 50°C for 24 hours (E24/50) and 37°C for 96 hours (E96/37). After the hydrolysis period, the resulting materials were equilibrated in a solution containing chlorine and alkaline sulfate (potassium, calcium and sodium). Excess salts were removed by washing in 3% (w/w) boric acid solution, deionized water, followed by 0.3% (w/w) EDTA solution, and finally in deionized water. The resulting material was equilibrated in pH 7.4 saline phosphate buffer for 24h and thoroughly washed with deionized water, frozen, lyophilized and kept in aseptic containers. Prior to implantation, the dies were cut into discs, Ø = 3 mm and hydrated in sterile saline for 24 h (Fig 1A1).

#### Characterization of the elastin matrix

*Differential scanning calorimetry–DSC*. The temperature of thermal denaturation (Td) was determined by differential scanning calorimetry (DSC-2010, TA Instruments, New Castle, USA). Measurements were made with approximately 18 mg of sample in airtight aluminum pans under a nitrogen atmosphere (80 mL / min), heating rate of 10°C min-1 and a temperature range of 5 to 120°C. The temperature of thermal denaturation was obtained at the point of inflection of the DSC curves.

#### Scanning electron microscopy–SEM

The photomicrographs were obtained on the matrix surface glued to stubs by conductive carbon tape and covered with a thin layer of gold 6 nm thick in a Coating System Baltec MED 020 metallizer (Leica Microsystems GmbH, Wetzlar, Germany) with pressure in the 2.00x10-2 mbar, current of 60 mA and deposition rate of 0.60 nms^-1^.

The matrix morphology was investigated by scanning electron microscopy (SEM) using a ZEISS LEO 440 instrument (Carl Zeiss MicroImaging GmbH, Jena, Germany) with an OXFORD 7060 detector (Oxford Instruments, Abingdon, United Kingdom), operating with an electron beam 20 kV.

The pore size was measured from the matrices using the Image JTM Program, (Java-based image processing program developed at the National Institutes of Health, Image J ^™^ 1.50d Wayne Rasband, National Institutes of Health, USA, Java 1.7_67; 64 -bit). 50 determinations were made for each matrix using an image enlarged 500 times [11] using Martin’s diameter approximation [12].

#### Synthetic hydroxyapatite

The hydroxyapatite evaluated in the present study is a microgranular ceramic, synthesized by the São Carlos Institute of Chemistry—USP, with particle size < 0.149 mm., Ca/P ratio obtained by EDX was 1.89 ± 0.04 and the major elements found were carbon (0.2 keV), oxygen (0.5 keV), calcium (3.7 and 4.0 keV), and phosphorus (2.0 keV) [13]. The sterilization of the biomaterial was by ethylene oxide gas. In the E24/50/HA and E96/37/HA groups, 0.05 mg of hydroxyapatite was deposited on the elastin matrices, an amount previously established in a pilot study (Fig 1A1).

#### Recombinant human/mouse/rat BMP-2 protein

The BMP used was the recombinant human BMP-2 (R&D System Inc., Minneapolis, MN, USA), source Chinese Hamster Ovary cell line, CHO - derived BMP-2 protein Gln283-Arg396; Structure/Form Disulfide-linked homodimer; Activity Measured by its ability to induce alkaline phosphatase production by ATDC5 mouse chondrogenic cells; Purity > 95%, by SDS-PAGE under reducing conditions and visualized by silver stain. Constructs with BMP-2 were prepared by adding 2 μg of rhBMP-2 in 0.0533 mL sterile 4 mM HCl containing at least 0.1% human or bovine serum albumin, resulting in a final concentration of 37.5 μg/mL, according to the manufacturer. This amount was chosen based on previous studies showing that 2–5μg BMP-2 is sufficient to stimulate bone regeneration in femur bone defect in rats [14,15].

Proteins were applied under the elastin matrices to the bone defect in the volume of 5 μL with an eppendorf^TM^ micropipette of 0.5 to 10 μL. Protein concentration was determined on the basis of the volume of the defect and applied as the amount necessary for its completion [16].

#### Experimental design

Seventy-seven male rats, *Rattus norvegicus*, Wistar, 120 days old and average weight of 320 grams (Fig 1A2) were used. The animals were supplied by the Institute of Energy and Nuclear Research (São Paulo—SP) and kept in the vivarium of the Jundiaí Medical School (FMJ). All animals were housed in boxes with a maximum of 3 animals each and received balanced feed (Purina) and water *ad libitum*. The environment was maintained with controlled temperature (23 ± 1°C) and light and dark cycle every 12 hours.

All experimental animal procedures were conducted with the approval of the Animal Research Ethics Committee of the Jundiaí Medical School (CEUA / FMJ), protocol 61/2015.

The animals were randomly divided into 7 groups: CG n = 11 (femoral defects filled with blood clot); E24/50 n = 11 (elastin matrix filled femoral defects hydrolysed for 24h at 50°C; E24/50/HA n = 11 (elastin matrix filled femoral defects hydrolyzed for 24h at 50°C and 0.05 mg hydroxyapatite; E24/50/BMP n = 11 (elastin matrix filled femoral defects hydrolyzed for 24h at 50°C and 5μl BMP; E96/37 n = 11 (elastin matrix filled femoral defects hydrolyzed for 96h at 37°C; E96/37/HA n = 11 (elastin matrix filled femoral defects hydrolyzed for 96h at 37°C and 0.05 mg hydroxyapatite; and E96/37/BMP n = 11 (elastin matrix filled femoral defects hydrolyzed for 96h at 37°C and 5μl BMP (Fig 1A2).

#### Bone defect surgeries

Animal surgeries were performed under general anesthesia with intramuscular injection into the buttock with ketamine (50 mg/kg im, Dopalen ^™^, Ceva, SP, Brazil), and xylazine (10 mg/kg im, Coopazine ^™^, Coopers, SP, Brazil), at a ratio of 1: 1 (0.10 mg/100 g body mass) [17]. Rats were positioned on the operation table in lateral recumbency with the right leg facing upwards. The lower right limb was trichotomized and aseptically prepared.

A medial parapatellar incision was performed, followed by arthrotomy and lateral patellar dislocation for better visualization of the distal femur region. The periosteum was carefully divulsed and then with the knee flexed, the femoral condyles were exposed (Fig 1B1). With the help of a trephine drill (Härte Precision GripTM, São Paulo, Brazil) at low speed coupled to the BeltecTM LB100 micromotor (Beltec Micromotors, SP, Brazil) an anteroposterior monocortical defect (φ = 3 mm) was created approximately below the growth plate and perpendicular to the shaft axis in the metaphyseal region of the distal femur with constant saline irrigation to avoid thermal necrosis (Fig 1B2).

The drilled holes were rinsed by injection with saline solution in order to remove bone fragments from the cavity (Fig 1B3). Bone grafts were then gently placed to fill the drilled defects according to group allocation, except for the CG, which remained empty (Fig 1B4). Afterwards, muscle tissue and skin were sutured separately.

The postoperative care consisted of a single intramuscular injection of pentabiotic at a dose of 0.1 mg/100g i.m (Fort Dodge^TM^, SP, Brazil), local application of rifamycin sodium spray (Rifocina^TM^, Sanofi-Aventis Pharmaceutical Ltda, SP, Brazil) and oral administration of acetaminophen at a dose of 200 mg/kg (Paracetamol, Medley, Sao Paulo, Brazil) dissolved in water, available in the cages for 14 days.

The animals were euthanized 42 days after the surgical procedure with excessive dose of xylazine and ketamine, followed by pneumothorax induced by sectioning of the diaphragm through the abdominal cavity. Next, the hip and knee disarticulations were performed with the removal of soft tissues and removal of the right femur.

Rat bone samples were separated according to the desired stain, calcification status, and embedding medium. Four animals/group followed the protocol for confocal laser scanning microscopy and the Von Kossa method and seven animals/group for macroscopic, radiological, micro CT, Masson trichrome staining and picrosirius red.

#### Sequential fluorescent labeling

Four animals from each experimental group underwent dorsal subcutaneous administration of fluorochromes in the postoperative period. Alizarin Red S 30 mg/kg injections (Sigma-AldrichTM, Merck KGaA, Darmstadt, Germany) were performed in the immediate postoperative period and 7 days after the surgical procedure. Calcein 10 mg/kg (Sigma-AldrichTM, Merck KGaA, Darmstadt, Germany) injections were performed at 14 and 21 days after the surgical procedure. All dyes were prepared immediately before use with disodium phosphate and saline solution.

#### Radiological analysis

Femur samples were collected and fixed in 10% phosphate-buffered formalin for 48 hours. They were then radiographed with an Odel 300 mA, 100mA focus, time of 0.06 s and 40 kV radiation and digitalized by the Agfa system to assess bone failure integrity, adjacent areas, and bone repair.

#### Micro‐CT Scan (μ‐CT)

The specimens were subjected to an X-ray beam scan in the SkyScan 1174v2 computed micrographograph machine (μ-CT-Bruker-microCT, Kontich, Belgium). The X-ray system is based on a micro focus tube (50 kV, 800 μA) generating projection images irradiating X-rays with cone beam geometry. The samples were positioned on a computer controlled rotation stage and scanned 180° around the vertical axis in rotation steps of 0.73° at 50 kV. The images were captured with 16.61 μm and further reconstructed using the NRecon^TM^ v.1.6.8.0 program (SkyScan, 2011, Bruker-microCT), with the same reconstruction parameters for all specimens. Next, the reconstructed images were realigned using the DataViewer^TM^ 1.4.4.0 software resulting in two-dimensional transaxial and sagittal images with 16 bits grey scale resolution. Next, images were reconstructed three-dimensionally using CTvox.

#### Histologic preparation for confocal laser scanning microscopy analysis

The specimens were fixed in 10% buffered formal for 2 days. They were sequentially dehydrated at increasing concentrations of 70% to 100% alcohols, soaked in glycol methacrylate resin, and polymerized. After polymerization, cross-sectional and semi-serial histological sections of 300 μm thickness were made and subsequently reduced with the aid of an automatic sander to 30 μm by the Exakt CuttingGrinding system (EXAKT Apparatus GmbH, Norderstedt, Germany). The sections were analyzed using a TCS SP5AOBS laser scanning confocal microscope (Leica^TM^, Wetzlar, Germany), coupled with a DFC 310 FX camera (Leica^TM^, Wetzlar, Germany), and QWin 3.1 (Leica^TM^, Wetzlar, Germany). The photomultiplier for each of the fluorescence markers was 488 nm (calcein) and 543 nm (alizarin red).

#### Histologic preparation and Von Kossa’s staining method

Von Kossa staining was applied to detect the presence of phosphate, an indicator of extracellular matrix mineralization. The preparation of slides for the Von Kossa staining method followed the same protocol as the confocal laser scanning microscopy analysis. The slides were washed three times in distilled water and immersed in 1% silver nitrate solution under ultraviolet radiation for 1 hour. After this process, they were immersed in 5% sodium thiosulfate solution and immediately washed in distilled water. This process gives the pink and black colorations, referring to the cytoplasm and calcium ions, respectively. Cell nuclei were counterstained by immersion for 5 minutes in safranin solution and glacial acetic acid. The slides were observed under a Motic BA310E series optical microscope (Motic^TM^, Kowloon, Hong Kong) and images captured on 4x and 10x lenses.

#### Histological procedures—Masson’s trichrome staining and picrosirius red

**Fig 2:**
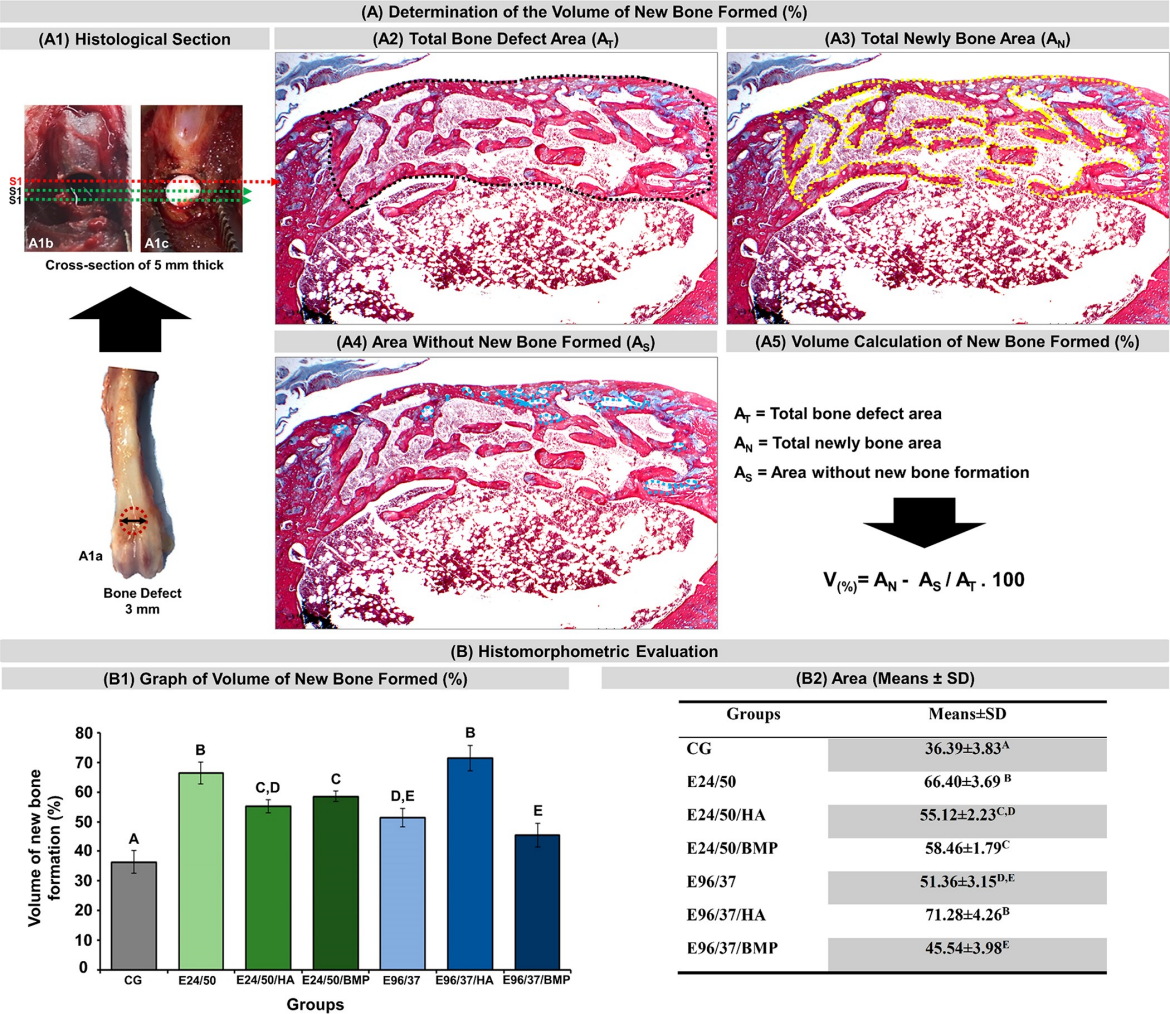
(A) Volume calculation of new bone formed (%) of all experimental groups. (A1) Defect of 3mm in diameter in the anterior metaphyseal region of the distal femur (A1a), defects filled with blood clot (A1b) and elastin matrix (A1c) show the location near the three 5μm cross-sectional sections obtained every 1250μm apart; black dashed line delimiting the total area of the bone defect (A2); yellow dashed line delimiting the area of newly formed bone (A3); blue dashed line delimiting the areas without new formed bone (A4); volume equation of new formed bone (A5). (**B**) Histomorphometric Evaluations: Graph of the volume of new bone formed (%) in each experimental group at 42 days (B1). Table of percentages of new bone formed from experimental groups (B2). Analysis was performed between the different experimental groups (GC, E24/50, E24/50/HA, E24/50/BMP, E96/37, E96/37/HA, E96 /37/BMP). ANOVA a criterion followed by the Tukey`s test. Mean ± standard deviation (n = 11 animals/group), where different letters (A ≠ B ≠ C ≠ D ≠ E) indicate statistically significant differences (p <0.01).

After micro-CT scanning, femur samples were decalcified in decalcifying acid Allkimia^TM^ (Allkimia Trade Materials Laboratory Ltd, Sao Paulo, Brazil) for 20 days. After this period, the acid action was neutralized for 24 hours in a 5% sodium sulfate solution. Sequentially, the specimens were histologically processed for embedding in Histosec^TM^ (paraffin enriched with polymers of the EMD Millipore—division of Merck KGaA, Darmstadt, Germany). Semi-serial cross sections of 5 μm thick (1250 μm intervals) of all defects were performed, collected on slide and stained with Masson's trichrome and picrosirius red (Fig 2A1a, 2A1b and 2A1c). The slides were observed under light microscope and polarized light for determining the quality of the newly formed organic matrix.

The histological analysis of the Masson's trichrome stained sections consisted of the evaluative description of the bone repair process for each treatment. Motic BA310E series microscope (MoticTM, Kowloon, Hong Kong) was used and digital images were analyzed with 4x and 40x objectives.

#### Histomorphometric evaluation

Masson's trichrome x4 objective digital images were used to quantify newly formed bone volume using the Motic Images Plus 2.0 software (Motic Digital Microscopy, Kowloon, Hong Kong). Bone defect margins and cortical thickness were marked with a black dashed line to identify the total lesion area (AT), the area of bone neoformation with a yellow line (AN) and the area without formation of new bone tissue, gaps with a blue line (AS) (Fig 2A2–2A4). The percentage of neoformation volume was obtained through the formula: V_(%)_ = A_N_—A_S_ / A_T_ x 100 (Fig 2A5).

### Statistical analysis

Data regarding the percentage of bone neoformation volume of the surgical area of each rat were transcribed to the BioEstat 5.3 software, applying the ANOVA tests followed by the Tukey`s test for statistical evaluation and obtaining means and standard deviations between the study groups with a significance level set at p <0.01.

## Results

### Characterization of the elastin matrix

#### Differential scanning calorimetry

**Fig 3:**
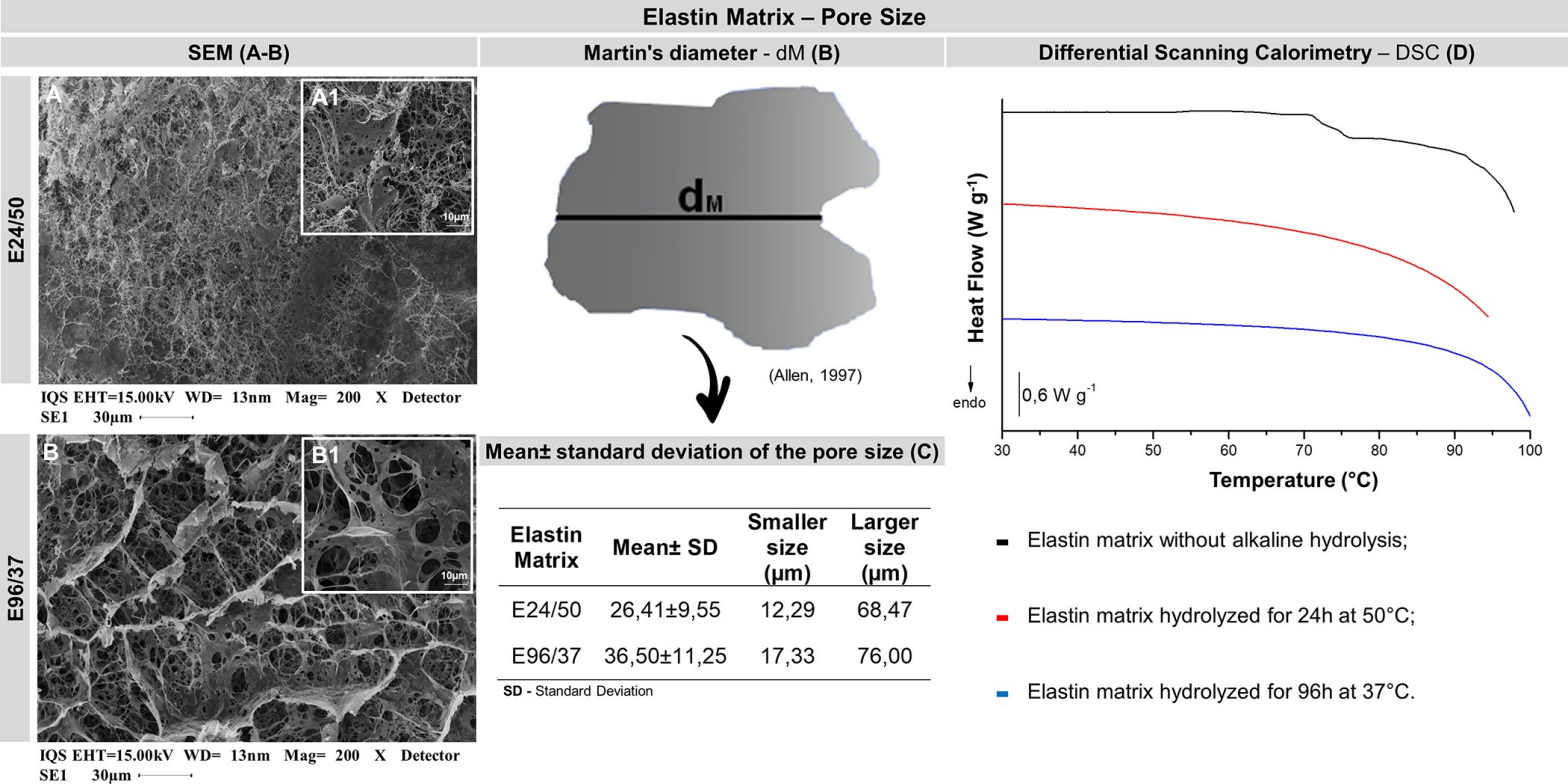
Elastin matrix–pore size. (**A, B**) Scanning electron microscopy images of elastin matrix surfaces in different magnifications 200x and 1000x, respectively. (**B**) The pore diameters were measured in SEM photomicrographs using an approximation of the diameter of Martin [12]. (**C**) Table of the mean + standard deviation of the pore size of the elastin matrices E24/50 and E96/37 and the sizes of the smallest and largest pores. SEM image 1000x magnification [11]. (**D**) Differential Scanning Calorimetry (DSC) curves of elastin matrices with or without alkaline hydrolysis.

The DSC curves did not show a transition in the temperature range studied, suggesting that there is no presence of collagen in the matrices, due to the long period of exposure of biological tissue, 96h to alkaline hydrolysis at a temperature of 37°C or even to the short interval, 24h, but with high temperature, 50°C. For the tissue without alkaline hydrolysis, a thermal transition at 75.2°C was observed, referring to the denaturation of the collagen structure present in the biological tissue (Fig 3D).

#### Scanning electron microscopy–SEM

Scanning electron microscopy images showed a rough surface, with pore sizes ranging from 12.29 to 68.47 μm and 17.33 to 76.0 μm E24 / 50 and E96 / 37, respectively disorganized (Fig 3A and 3B).

### Macroscopic and radiographic analysis

There were no obvious signs of morbidity, animal mortality and infection in the wound area or elsewhere after the surgical procedure. All animals presented good general conditions during the experimental period.

**Fig 4:**
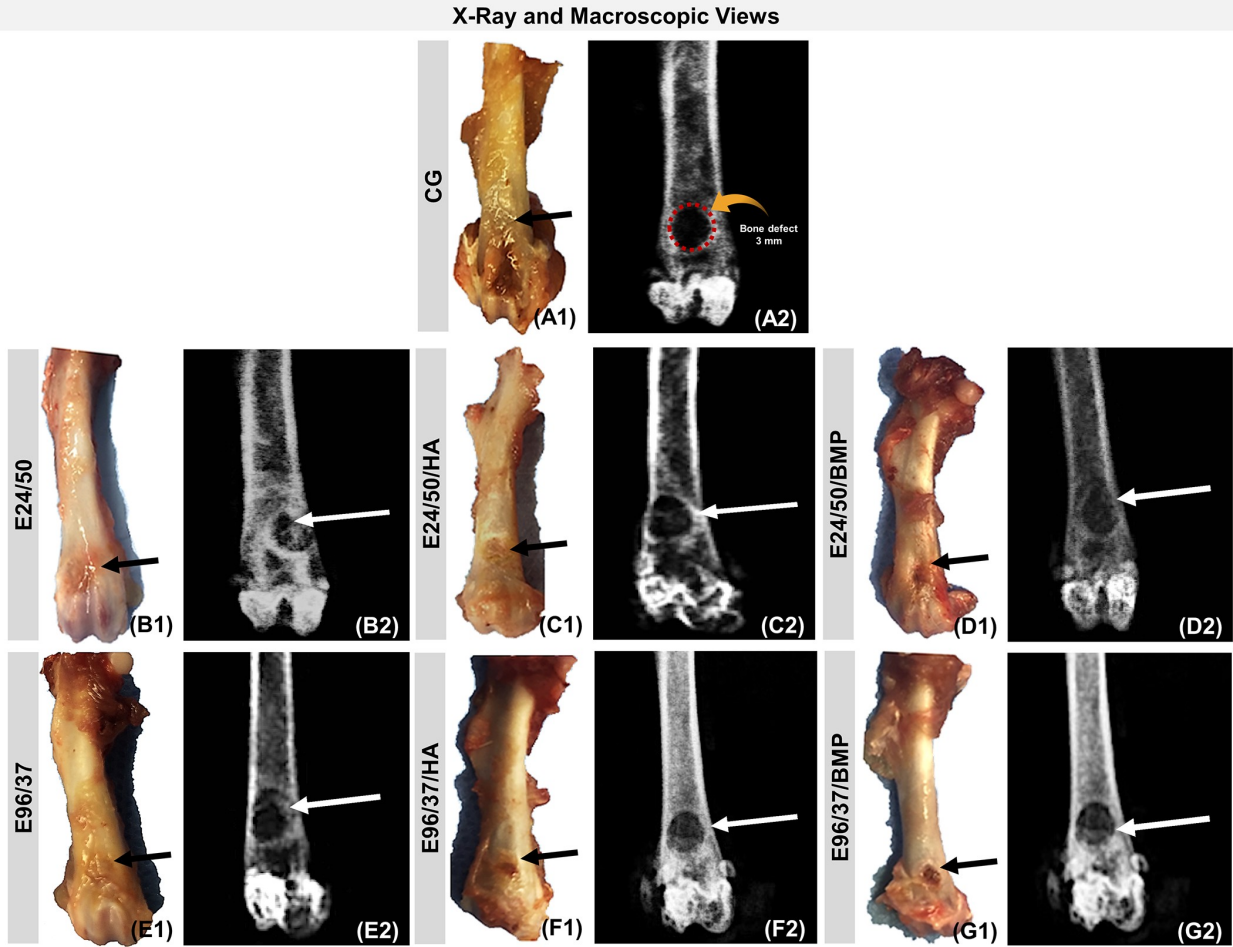
Evaluation of macroscopic features (A1-G1) and X-ray (A2-G2) of bone defects in the anterior metaphyseal region of the distal femur of rats filled with blood clot, CG, (A1-A2); hydrolyzed elastin membrane for 24h at 50°C, E24/50, (B1-B2); hydrolyzed elastin membrane for 24h at 50°C and hydroxyapatite, E24/50/HA, (C1-C2); elastin membrane hydrolyzed for 24h at 50°C and BMP, E24/50/BMP, (D1-D2); elastin membrane hydrolyzed for 96h at 37°C, E96/37, (E1-E2); elastin membrane hydrolyzed for 96h at 37°C and hydroxyapatite, E96/37/HA, (F1-F2) and elastin membrane hydrolyzed for 96h at 37°C and BMP, E96/37/BMP, (G1-G2). The white and black arrows indicate the surgical area and the dashed circle in the CG group indicates the diameter of the bone defect of 3 mm.

From a superficial macroscopic analysis of the surgical area, it was observed in all animals that the soft tissue repair process occurred without complications or inflammatory reaction. After removal of the surrounding soft tissues, the longitudinal integrity of the femur was maintained without the presence of fractures or bone deformities. The margins of the bone defects were well delimited and the total surface area was covered by connective tissue (Fig 4A1–4G1).

Radiographically, in all specimens no bone thinning, signs indicative of osteonecrosis, or periosteal reactions suggestive of infectious or tumoral process were observed (Fig 4A2–4G2).

In the CG control group images, a radiolucent cavity corresponding to the removed bone fragment was laterally delimited by the radiopaque bone edge (Fig 4A2).

In the treated defects, the E24/50/HA and E96/37/HA groups presented radiopacity in bone failures indicating remnants of hydroxyapatite particles and / or newly formed bone tissue (Fig 4C2–4F2). The remaining defects in the E24/50, E24/50/BMP, E96/37 and E96/37/BMP groups showed formation of new bone tissue evidenced by radiopaque images inside the surgical cavities (Fig 4B2, 4D2, 4E2 and 4G2).

### Micro CT analysis—three-dimensional and two-dimensional sections

**Fig 5:**
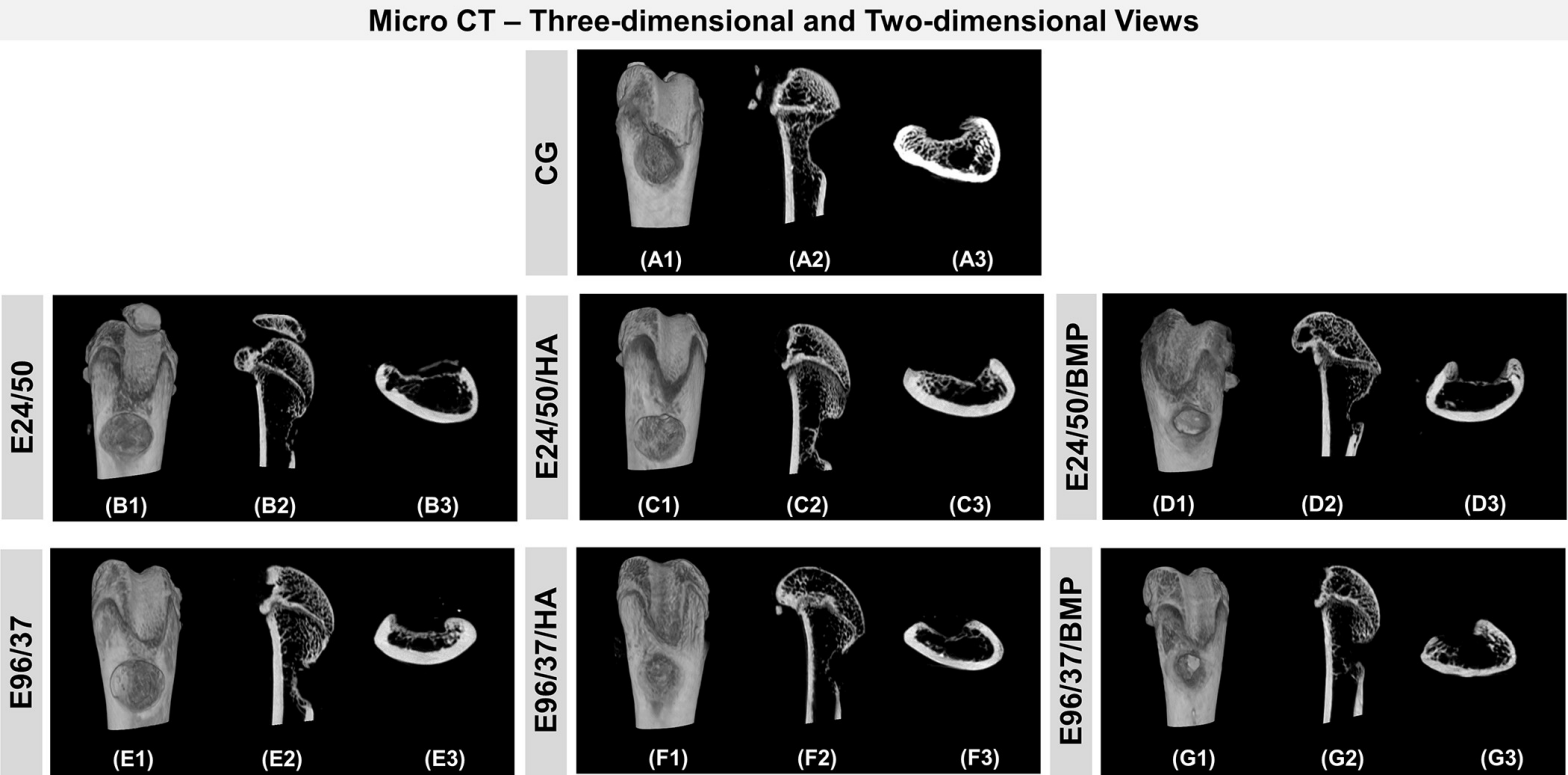
Micro CT assessments. A) Three-dimensional reconstruction area: shows metaphyseal defects of the distal femur of the CG mice (unfilled), and groups filled with E24/50, E24/50/HA, E24/50/BMP, E96/37, E96/37/HA and E96/37/ BMP (A1-G1, respectively). B) Two-dimensional view: Representative sagittal (A2-G2) and transaxial (A3-G3) reconstruction images obtained by the micro-CT analysis of femoral rat bone from the CG, E24/50, E24/50/HA, E24/50/BMP, E96/37, E96/37/HA and E96/37/BMP groups.

Six weeks (42 days) after implantation of the biomaterials, microtomography images demonstrated healthy bone formation in all experimental groups. No treated defect detected the presence of elastin matrices (Fig 5).

The described analysis of the transaxial and sagittal sections of the CG group showed partial closure by mineralized tissue, which was confirmed by histological analysis (Fig 5A2 and 5A3). Only in the treated defects of the E96/37/HA groups, did a thin, compact bone bridge form the interconnected edges of the surgical area with hyperdensity similar to intact cortical bone (Fig 5F2 and 5F3).

In the three-dimensional images, all defects had concavity in the cortical surface, being less pronounced in the E96/37/HA group (Fig 5A1–5G1).

Defects filled with the elastin and hydroxyapatite matrices, the E24/50/HA and E96/37/HA groups revealed some spots within the defect, characterized by the high density, similar to the remaining bone tissue, related to the hydroxyapatite microgranules (Fig 5C1–5F1).

### Confocal laser scanning microscopy analysis

**Fig 6:**
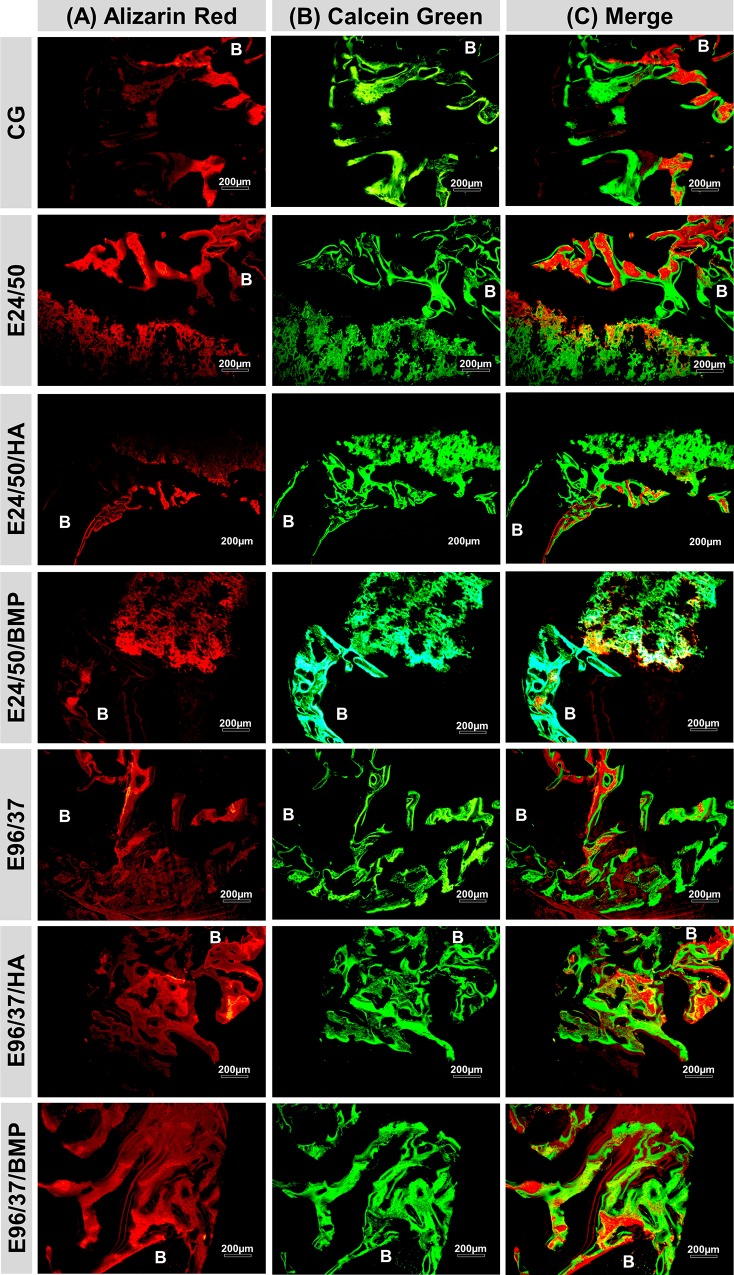
Confocal laser scanning microscopy group images (CG, E24/50, E24/50/HA, E24/50/BMP, E96/37, E96/37/HA and E96/37/BMP). The different colors show the bone formation at different time periods. (A) Alizarin red applied in the immediate postoperative period and 7 days after the surgical procedure. (B) Calcein green applied at 14 and 21 days after surgical procedure. (C) Folded image of the two fluorochromes. Remaining bone (B). Objetive x20, Bar: 200 μm.

Fluorescent markers, alizarin red and calcein were used to qualitatively assess bone mineral apposition rates (Fig 6A–6C).

The images obtained demonstrated red and green areas that are potentially identified as fluorochrome-marked calcium precipitation regions at different times of tissue mineralization.

In all experimental groups, the mineralization sites showed similar characteristics with areas strongly marked by alizarin fluorochrome, close to the remaining cortical bone and the periosteal surface, in contrast to large areas without fluorochrome marking, dark field and calcein deposit in the central region, underlying the bone defect.

### Mineralization analysis of new bone formed by the Von Kossa’s method

**Fig 7:**
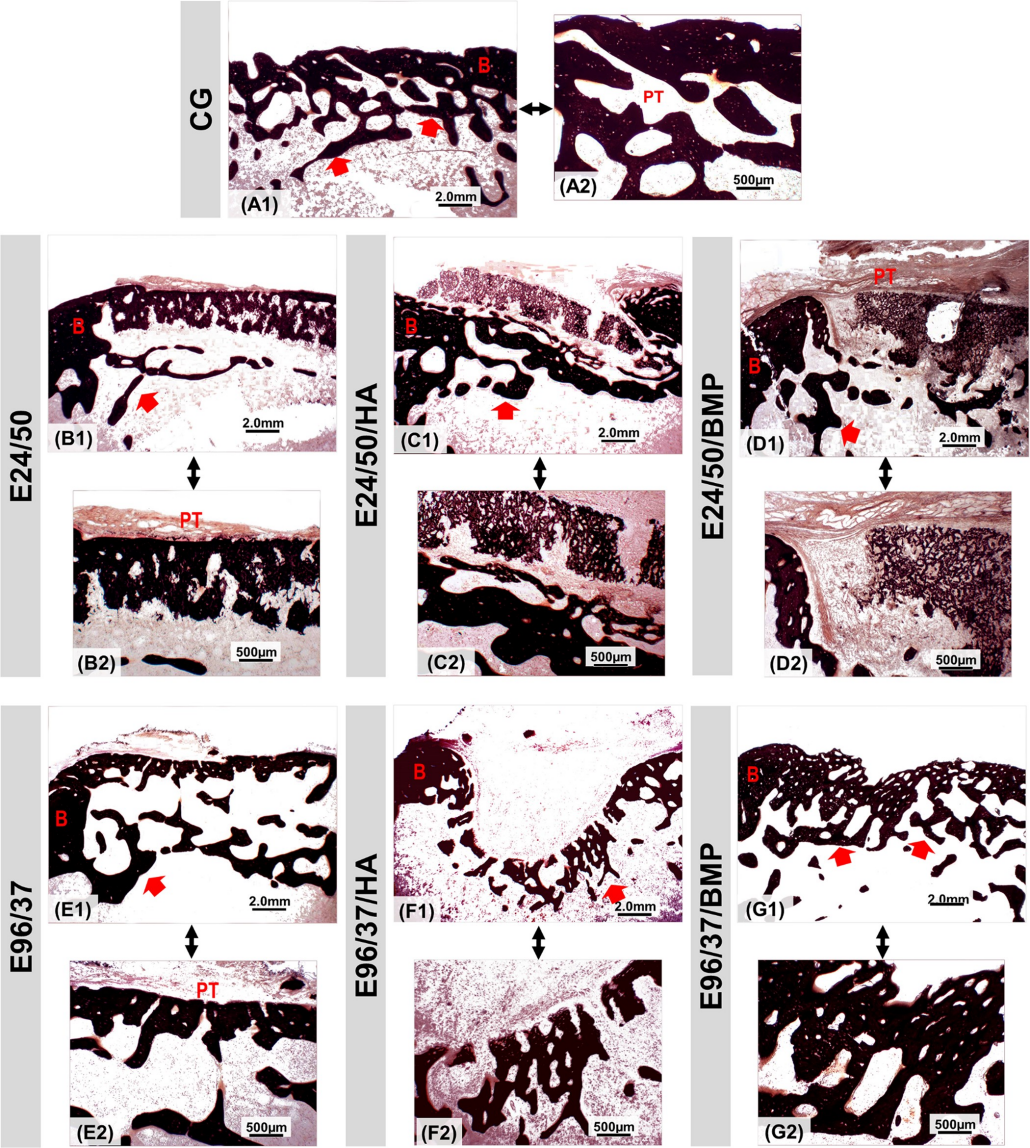
Von Kossa stained section at the metaphyseal region of the distal rat femur at 4x (A1-G1) and 10x (A2-G2) magnifications for groups: E24/50, E24/50/HA, E24/50/BMP, E96/37, E96/37/HA e E96/37/BMP. Periosteal tissue (PT); trabecular plate (red arrow); surgical site border (B). Bar: 2.0 mm and 500 μm.

The sections stained by Von Kossa revealed the presence of a black mineralized thin layer with discontinuous trabecular formation in the cortical space in all experimental groups (Fig 7).

### Birefringence analysis of collagen fibers

**Fig 8:**
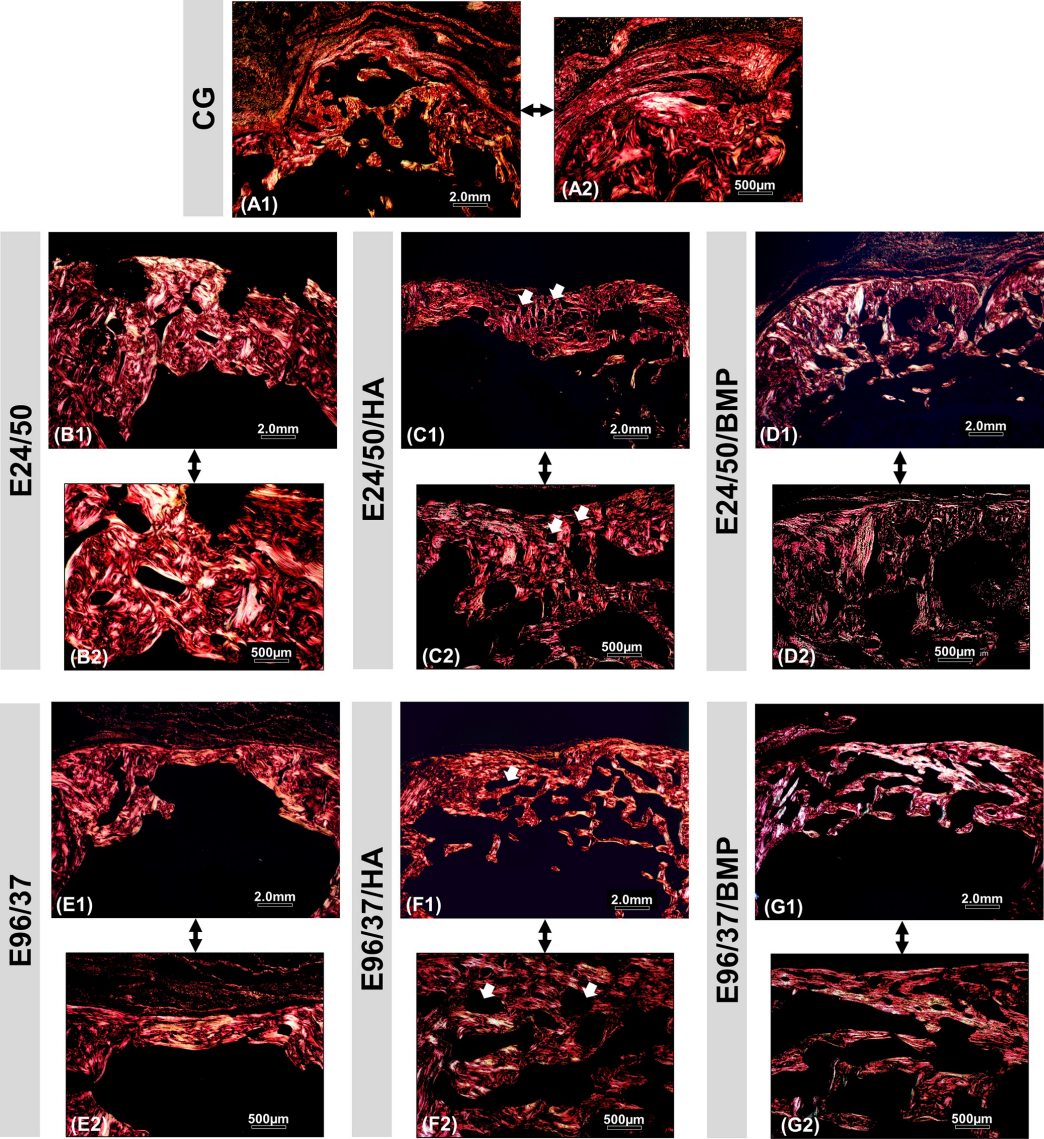
Images of birefringent collagen fibers by PSR-polarization method in the area of bone defect created in the distal femoral metaphysis in rats. Picrosirius red (PSR) stained collagen fibers are specifically birefringent in polarized light, fine green/type III fibers; thick red fibers/type I. Red-orange birefringence was observed predominantly in all experimental groups at 42 days, corresponding to thicker and more organized collagen fibers. Note the similarity between the birefringence of the remaining collagen fibers of the newly formed tissue. Hydroxyapatite particle (white arrow). Original 4x magnification: (A1-G1) Bar: 2 mm. Inset10x magnified images (A2-G2) Bar: 500 μm.

Representative images of picrosirius-red staining revealed a similar predominance of red-orange birefringence in all experimental groups in the cortical region, corresponding to the thicker collagen fibers, type 1. In defects treated with elastin and hydroxyapatite matrices, E24/50/HA and E96/37/HA, the biomaterial particles appeared in a dark field surrounded by collagen fibers arranged with red-orange birefringence (Fig 8C1, 8C2, 8F1 and 8F2).

### Histological evaluation of regenerated bone

**Fig 9:**
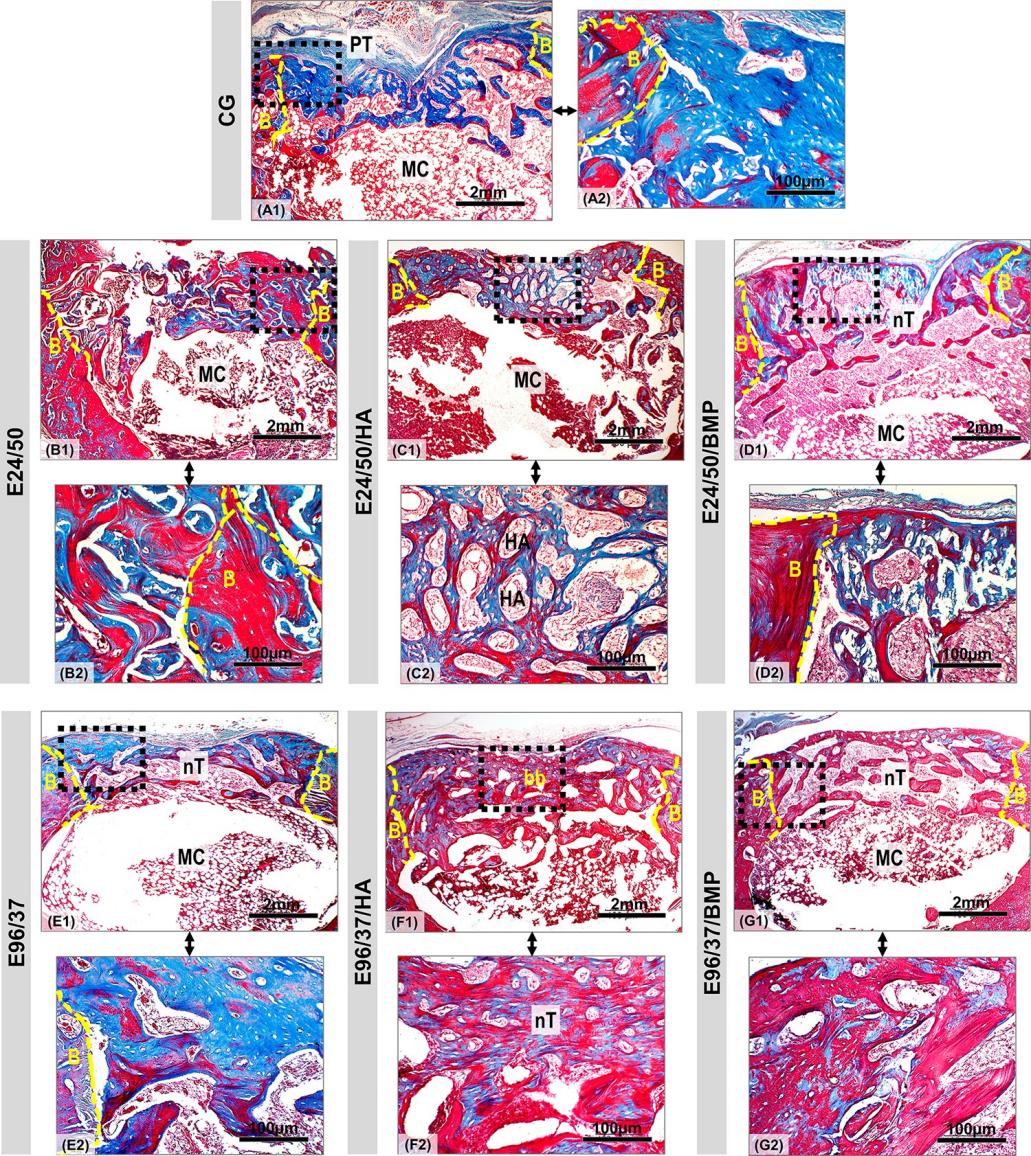
Panoramic view of bone defect created in distal femoral metaphysis in rats. Defect area in the CG, E24/50, E24/50/HA, E24/50/BMP, E96/37, E96/37/HA and E96/37/BMP (A1-G1) experimental groups at 42 days. All experimental groups presented trabecular bone formation from the remnant cortical border in transition from immature to mature bone, but without complete healing of the surgical wound. In the E96/37/HA treated defect, the bone trabeculae were more mature, thicker and more organized, forming a bone bridge connecting the defect edges. Masson's trichrome staining: new bones with blue dye and mature bones with red dye. Periosteal tissue (PT); defect border (B); medullary canal (MC); neoformed bone trabeculae in the cortical region (nT); bone bridge (bb); Hydroxyapatite particle (HA). Masson's trichrome staining of defect area. Original 4x magnification, Bar: 2mm. Inset 40x magnified images (A2-G2). Bar: 100 μm.

Histological analysis of the CG group defects at 42 days after surgery showed mineralized bone formation at the edges of the defect and adjacent to the periosteal tissue, but without complete cortical restoration. In the underlying cortical region, the bone tissue showed trabeculated conformation and the medullary canal was preserved (Fig 9A1 and 9A2).

The E24/50, E24/50/HA, E24/50/BMP, E96/37, E96/37/HA, E96/37/BMP treated defects showed no signs of inflammation, foreign body reaction, or fibrous connective tissue in the deeper areas and near the cortical spaces (Fig 9B2–9G2). Bone repair was partial, as indicated by the presence of areas without bone formation that were invaded by connective tissue (Fig 9B1–9G1).

The newly formed bone matrix in all groups exhibited mature trabecular features harboring osteocytes in continuity with the central failure region (Fig 9A2–9G2). The most pronounced bone neoformation occurred in the E96/37/HA group, such that it allowed the formation of a thin bone bridge joining the margins of the remaining bone (Fig 9F1).

In the E24/50 /HA and E96/37/HA groups, hydroxyapatite particles surrounded by newly formed bone tissue were noted (Fig 9C2–9F2).

### Histomorphometric evaluation

In the quantitative evaluation of the relative percentage volume of newly formed bone, it was observed that all defects filled with biomaterial, E24/50, E24/50/HA, E24/50/ BMP, E96/37, E96/37/HA, E96/37/BMP (66.40 ± 3.69; 55.12 ± 2.23; 58.46 ± 1.79; 51.36 ± 3.15; 71.28 ± 4.26 and 45.54 ± 3.98, respectively) had higher means with significant difference compared to the CG group (36.39 ± 3.86) (Fig 2B1 and 2B2).

The highest percentages of newly formed bone volume were observed in the E96/37/HA and E24/50 groups (71.28 ± 4.26 and 66.40 ± 3.69, respectively) but were not significantly different from each other (Fig 2B1 and 2B2).

In the comparative analyses of the mean percentage of newly formed bone between the E24/50/HA (55.12 ± 2.23) vs. E24/50/BMP (58.46 ± 1.79), E24/50/HA (55.12 ± 2.23) vs. E96/37 (51.36 ± 3.15) and E96/37 (51.36 ± 3.15) vs. E96/37/BMP (45.54 ± 3.98) groups showed no significant difference between them, unlike the other comparisons with p <0.01 (Fig 2B2).

## Discussion

The increase in bone disease rates triggered by fractures, pseudoarthrosis, osteoporosis and or tumor resection has aroused interest in the development of new biomaterials in order to restore the morphological and functional integrity of the lost tissue [18, 19].

Although it is evident that the elastin matrix combines favorable clinical management properties with positive biological response in bone tissue repair, there is still a lack of scientific studies when associated with growth factors and osteoconductive materials [20].

Thus, the present study provides evidence that hydrolyzed elastin matrix at 37°C/96h loaded with hydroxyapatite crystals and hydrolyzed elastin matrix at 50°C/24h accelerated the repair process in metaphyseal bone defects in rat femurs.

In recent years, the metaphyseal drill hole defects in the femur model adopted in the present study has been a focus of great interest in preclinical research since it provides highly standardized bone repair conditions, a favorable model for testing biofunctional properties of scaffolding, without the need for limb immobilization or fixation and low risk of postoperative fracture [1, 21–23].

To investigate the performance of hydrolyzed elastin matrices associated or not with synthetic hydroxyapatite and bone morphogenetic protein, we used macroscopic, radiographic, microtomographic analysis, laser confocal microscopy, Von Kossa staining, histological, histomorphometric and birefringence analysis of collagen fibers.

From a superficial macroscopic analysis of the surgical area, all animals presented longitudinal integrity of the femur, without the presence of fractures, confirmed by radiographic images. These findings may be related to the biomechanical stability of the metaphyseal bone defect model which decreases the incidence of postoperative complications and consequently fractures [24–26].

In the two-dimensional and three-dimensional reconstructions obtained by μCT, the experimental groups presented mineralized bone trabeculae at the margins of the ruptured cortical region. However, in the E96/37/HA group, a thin, hyperdense layer was observed interconnecting the defect area. This finding may be justified by the nucleation of hydroxyapatite crystals at specific locations of the elastin matrix by bonding with calcium ions. Thus, the supersaturation of physiological fluids in phosphate ions causes precipitation of the mineral phase and progressively extends along the elastin fibrils [27, 28].

Histologically, in the unfilled defects, CG, bone formation was initiated in the deepest region of the defect from pre-existing trabeculae and spread toward the cortical space. Cortical bone formation was slow and incomplete because it failed to regenerate the metaphyseal cortex observed in normal mature bone at 42 days after surgery [29]. Failure to regenerate these bone defects is supported by preliminary studies that claim to be critical size defects requiring reconstructive procedures [11, 25, 30–32].

All defects filled with biomaterials showed no chronic inflammatory response or foreign body reaction, but biocompatible interactions with molecular and cellular events occurring in a natural cellular microenvironment [33]. This fact can be explained by the chemical processes of alkaline hydrolysis used for the fabrication of the elastin matrix derived from bovine auricular cartilage permit the removal of impurities and remnant cells, thus preventing any type of immune rejection of the collagen when implanted into the host tissue.

This data can also be corroborated by the differential exploratory calorimetry analysis that evidenced the denaturation of the collagen triple helix structure of the elastin matrices, E24/50 and E96/37, since the integrity of the collagen fibers present thermal transition at 75.2°C.

Histomorphometrically, the E96/37/HA and E24/50 groups presented the highest average of new bone volume formed (71.28 ± 4.26 and 66.40 ± 3.69, respectively) with a statistically significant difference compared to the other experimental groups: CG, E24/50/HA, E24/50/BMP and E96/37, E96/37/BMP (36.39 ± 3.83, 55.12 ± 2.23, 58.46 ± 1.79, 51.36 ± 3.15 and 45.54 ± 3.98, respectively), but without statistical significance to each other.

Previous investigations justify these results since they report that the alkaline hydrolysis process at a given reaction time and temperature may have influenced the variation of the surface morphology of the elastin matrices, changing the spatial organization of the fibrils and their surface topography [5, 33, 34].

Thus, it is suggested that the hydrolyzed elastin matrix at 50°C/ 24 hours presented a cellular microenvironment that mimics the characteristics of the native extracellular matrix with a peptide sequence that favors binding with integrins of many cell types, influencing signaling, chemotaxis and cell proliferation [6, 11]. However, the surface morphology of hydrolyzed elastin matrices at 37°C/ 97 hours did not favor cell growth when used alone [35].

Another factor that may have influenced the porosity of these biomaterials, that is, the incorporation of hydroxyapatite crystals into the hydrolyzed elastin matrix at 37°C/ 97h may have facilitated the interconnectivity between the micros and macro-pores, allowing the exchange of body fluids, addition of ions and vascular and cellular proliferation, unlike what was observed with the association of hydrolyzed elastin matrix at 50°C/ 24 hours and bio-ceramics [36].

When comparing the E96/37/BMP vs. E24/50/BMP groups (45.54 ± 3.98 vs. 58.46 ± 1.79, respectively) with the E96/37 vs. the E24/50 groups (71.28 ± 4.26 vs. 66.40 ± 3.69, respectively), lower mean volume of new bone formed in defects filled with bone morphogenetic protein matrices is observed. This finding can be attributed to inflammatory cells that adhere to and spread across the surface of the elastin fibrils and release degradation mediators such as reactive oxygen species, proteolytic enzymes and acids (pH <4.0) that may have degraded the BMP and consequently dose threshold for bone induction [28, 29].

Given the results obtained in the confocal analysis, it was possible to observe that all groups presented new bone markings formed during the experimental period, but with dark field predominance, with no markings in the CG group. This finding confirms the osteoregenerative capacity of the biomaterials employed, since the newly formed bone volumes of all groups: E24/ 50, E24/50/HA, E24/50/BMP, E96/37, E96/37/HA and E96/37/BMP (66.40 ± 3.69, 55.12 ± 2.23, 58.46 ± 1.79, 51.36 ± 3.15, 71.28 ± 4.26 and 45.54 ± 3.98, respectively) were higher when compared to the control group (36.39 ± 3.83) [37].

Von Kossa staining revealed intense calcium deposits distributed in all experimental groups. These results demonstrate that hydroxyapatite or morphogenetic protein-loaded elastin matrices can be mineralized in order to mimic the intra and extrafibrillary mineralization profile of native bone, making it a favorable microenvironment for osteogenic proliferation and osteogenesis differentiation, as well as structural support for vascular growth [38].

Descriptive analysis of collagen fibers under polarized light showed a predominance of red-orange birefringence in newly regenerated bone referring to thicker and more organized fibers, similar to the original lamellar bone of the defect edge [39]. In the E24/50/HA and E96/37/HA groups, the hydroxyapatite microparticles were in darkfield surrounded by red-orange birefringence collagen fibers arranged neatly [32].

It was concluded that the hydrolysed elastin matrix, E24/50 and the association of the hydrolyzed elastin matrix, E96/37 with the hydroxyapatite crystals promoted the bone repair process in the distal femoral metaphysis of rats.

## Limitations and future perspectives

Each methodology used in experimental research has its own merits and limitations in relation to specific needs. Therefore, in this study, we can point out as possible limitations the failure to assess the ability of stem cells to adhere to the analyzed matrix and cell differentiation in vitro, in addition to immunohistochemical and immunofluorescence analyzes, making it potential prospective studies, as the elastin matrix still has few reports in the literature [40, 41].

Based on current results and the perspective of new analyzes of the performance of the bone substitute, with a view to reducing the time of effective functional recovery and reinforcing the translational potential of preclinical research, therapies such as pulsed ultrasound (LIPUS) and laser photobiomodulation therapy (PBMT) can be added, which can allow a significant impact in reducing health care costs [42–45].

## Supporting information

S1 Graphical abstract (TIF)

## References

[1] Cheng N, Wang Y, Zhang Y, Shi B. The osteogenic potential of mesoporous bioglasses/silk and non-mesoporous bioglasses/silk scaffolds in ovariectomized rats: In vitro and in vivo evaluation. PLoS One. 2013;8: 1–16. 10.1371/journal.pone.0081014 24265840 PMC3827187

[2] Echave MC, Burgo LS, Pedraz JL, Orive G. Gelatin as Biomaterial for Tissue Engineering. Curr Pharm Des. 2017;23: 3567–3584. 10.2174/0929867324666170511123101 28494717

[3] Fernandez de Grado G, Keller L, Idoux-Gillet Y, Wagner Q, Musset AM, Benkirane-Jessel N, et al Bone substitutes: a review of their characteristics, clinical use, and perspectives for large bone defects management. J Tissue Eng. 2018;9 10.1177/2041731418776819 29899969 PMC5990883

[4] Sohn HS, Oh JK. Review of bone graft and bone substitutes with an emphasis on fracture surgeries. Biomater Res. 2019;23: 4–10. 10.1186/s40824-018-0149-3 30915231 PMC6417250

[5] Zhao F, Wang J, Guo H, Liu S, He W. The effects of surface properties of nanostructured bone repair materials on their performances. J Nanomater. 2015;2015 10.1155/2015/127235

[6] Daamen WF, Veerkamp JH, van Hest JCM, van Kuppevelt TH. Elastin as a biomaterial for tissue engineering. Biomaterials. 2007;28: 4378–4398. 10.1016/j.biomaterials.2007.06.025 17631957

[7] Kim S, Lee S, Kim K. Bone Tissue Engineering Strategies in Co-Delivery of Bone Morphogenetic Protein-2 and Biochemical Signaling Factors. Adv Exp Med Biol. 2018;1078: 233–244. 10.1007/978-981-13-0950-2_12 30357626

[8] Blair HC, Larrouture QC, Li Y, Lin H, Beer-Stoltz D, Liu L, et al Osteoblast differentiation and bone matrix formation in vivo and in vitro. Tissue Eng Part B Rev. 2017;23: 268–280. 10.1089/ten.TEB.2016.0454 27846781 PMC5467150

[9] Zhu B, Xu W, Liu J, Ding J, Chen X. Osteoinductive Agents-Incorporated Three-Dimensional Biphasic Polymer Scaffold for Synergistic Bone Regeneration. ACS Biomater Sci Eng. 2019;5: 986–995. 10.1021/acsbiomaterials.8b01371 33405789

[10] Horn MM, Martins VCA, de Guzzi Plepis AM. Interaction of anionic collagen with chitosan: Effect on thermal and morphological characteristics. Carbohy Polym. 2009;77: 239–243. 10.1016/j.carbpol.2008.12.039

[11] de Moraes R, de Guzzi Plepis AM, da Conceição Amaro Martins V, Duarte MAH, Alcalde MP, Buchaim RL, et al Suitability of the use of an elastin matrix combined with bone morphogenetic protein for the repair of cranial defects. Am J Transl Res. 2019;11: 5261–5271. 31497239 PMC6731398

[12] Allen T. Particle Size Measurement- Powder sampling and particle size measurement. 5 ed Chapman & Hall 5 ed. 1997 p. 525.

[13] Munhoz MAS, Hirata HH, Plepis AMG, Martins VCA, Cunha MR. Use of collagen/chitosan sponges mineralized with hydroxyapatite for the repair of cranial defects in rats. Injury. 2018;49: 2154–2160. 10.1016/j.injury.2018.09.018 30268514

[14] Bessa PC, Balmayor ER, Hartinger J, Zanoni G, Dopler D, Meinl A, et al Silk Fibroin Microparticles as Carriers for Delivery. J Tissue Eng Regen Med. 2010;16: 937–945. 10.1089/ten.TEC.2009.0486 19958078

[15] Koolen MKE, Longoni A, Van Der Stok J, Van Der Jagt O, Gawlitta D, Weinans H. Complete regeneration of large bone defects in rats with commercially available fibrin loaded with BMP-2. Eur Cell Mater. 2019;38: 94–105. 10.22203/eCM.v038a08 31529455

[16] Machado EG, Issa JPM, Figueiredo FAT de, Santos GR dos, Galdeano EA, Alves MC, et al A new heterologous fibrin sealant as scaffold to recombinant human bone morphogenetic protein-2 (rhBMP-2) and natural latex proteins for the repair of tibial bone defects. Acta Histochem. 2015;117: 288–296. 10.1016/j.acthis.2015.03.006 25825118

[17] Cunha MR, Santos AR, Goissis G, Genari SC. Implants of polyanionic collagen matrix in bone defects of ovariectomized rats. J Mater Sci Mater Med. 2008;19: 1341–1348. 10.1007/s10856-006-0105-y 17914639

[18] Winkler T, Sass FA, Duda GN, Schmidt-Bleek K. A review of biomaterials in bone defect healing, remaining shortcomings and future opportunities for bone tissue engineering. Bone Jt Res. 2018;7: 232–243. 10.1302/2046-3758.73.bjr-2017-0270.r1 29922441 PMC5987690

[19] Pomini KT, Buchaim DV, Shindo JVTC, Flato UAP, Rosso MP de O, Andreo JC, et al Applicability of Homologous Fibrin Sealant in Bone Repair: An integrative Review. IJAERS. 2019;6: 16–23. 10.22161/ijaers.673

[20] Wang W, Yeung KWK. Bone grafts and biomaterials substitutes for bone defect repair: A review. Bioact Mater. 2017;2: 224–247. 10.1016/j.bioactmat.2017.05.007 29744432 PMC5935655

[21] Uusitalo H, Rantakokko J, Ahonen M, Jämsä T, Tuukkanen J, KäHäri VM, et al A metaphyseal defect model of the femur for studies of murine bone healing. Bone. 2001;28: 423–429. 10.1016/s8756-3282(01)00406-9 11336924

[22] Tao ZS, Zhou WS, Tu KK, Huang ZL, Zhou Q, Sun T, et al Effect exerted by Teriparatide upon Repair Function of β-tricalcium phosphate to ovariectomised rat’s femoral metaphysis defect caused by osteoporosis. Injury. 2015;46: 2134–2141. 10.1016/j.injury.2015.07.042 26306803

[23] Alt V, Cheung WH, Chow SKH, Thormann U, Cheung ENM, Lips KS, et al Bone formation and degradation behavior of nanocrystalline hydroxyapatite with or without collagen-type 1 in osteoporotic bone defects—An experimental study in osteoporotic goats. Injury. 2016;47: S58–S65. 10.1016/S0020-1383(16)47010-5 27338229

[24] Edgerton BC, An K, Morrey BF. Torsional Strength Reduction Due. J Orthop Res. 2004;8: 1–5. Available: papers2://publication/uuid/735DF379-C628-487A-8596-D8BFD69F3349 10.1002/jor.1100080610 2213342

[25] Monfoulet L, Rabier B, Chassande O, Fricain JC. Drilled hole defects in mouse femur as models of intramembranous cortical and cancellous bone regeneration. Calcif Tissue Int. 2010;86: 72–81. 10.1007/s00223-009-9314-y 19953233

[26] Pobloth AM, Johnson KA, Schell H, Kolarczik N, Wulsten D, Duda GN, et al Establishment of a preclinical ovine screening model for the investigation of bone tissue engineering strategies in cancellous and cortical bone defects. BMC Musculoskelet Disord. 2016;17: 1–12. 10.1186/s12891-015-0856-z 26932531 PMC4774005

[27] Tampieri A, Sprio S, Sandri M, Valentini F. Mimicking natural bio-mineralization processes: A new tool for osteochondral scaffold development. Trends Biotechnol. 2011;29: 526–535. 10.1016/j.tibtech.2011.04.011 21645938

[28] Amini AR, Laurencin CT, Nukavarapu SP. Bone tissue engineering: Recent advances and challenges. Crit Rev Biomed Eng. 2012;40: 363–408. 10.1615/critrevbiomedeng.v40.i5.10 23339648 PMC3766369

[29] Mantripragada V, Jayasuriya A. Bone regeneration using injectable BMP-7 loaded chitosan microparticles in rat femoral defect. Mater Sci Eng C Mater Biol Appl. 2016;63: 596–608. 10.1016/j.msec.2016.02.080 27040255 PMC4839977

[30] Guéhennec L Le, Layrolle P, Daculsi G. A review of bioceramics and fibrin sealant. Eur Cell Mater. 2004 10.22203/eCM.v008a01 15494929

[31] Schindeler A, Mills RJ, Bobyn JD, Little DG. Preclinical models for orthopedic research and bone tissue engineering. J Orthop Res. 2018;36: 832–840. 10.1002/jor.23824 29205478

[32] Pomini K, Cestari M, German I, Rosso M, Gonçalves J, Buchaim D, et al Influence of experimental alcoholism on the repair process of bone defects filled with beta-tricalcium phosphate. Drug Alcohol Depend. 2019 10.1016/j.drugalcdep.2018.12.031 30875652

[33] Chen F, Liu X. Advancing biomaterials of human origin for tissue engineering. Prog Polym Sci. 2016;53: 86–168. 10.1016/j.progpolymsci.2015.02.004 27022202 PMC4808059

[34] Langdon SE, Chernecky R, Pereira CA, Abdulla D, J Michael L. Biaxial mechanical/structural effects of equibiaxial strain during crosslinking of bovine pericardial xenograft materials. Biomaterials. 1999;20: 137–153. 10.1016/s0142-9612(98)00142-2 10022783

[35] Sivaraman B, Bashur CA, Ramamurthi A. Advances in biomimetic regeneration of elastic matrix structures. Drug Deliv Transl Res. 2012;2: 323–350. 10.1007/s13346-012-0070-6 23355960 PMC3551595

[36] Velasco MA, Narváez-Tovar CA, Garzón-Alvarado DA. Design, materials, and mechanobiology of biodegradable scaffolds for bone tissue engineering. Biomed Res Int. 2015;2015 10.1155/2015/729076 25883972 PMC4391163

[37] Oryan A, Alidadi S, Moshiri A, Maffulli N. Bone regenerative medicine: Classic options, novel strategies, and future directions. J Orthop Surg Res. 2014;9: 1–27. 10.1186/1749-799X-9-1 24628910 PMC3995444

[38] Thrivikraman G, Athirasala A, Gordon R, Zhang L, Bergan R, Keene DR, et al Rapid fabrication of vascularized and innervated cell-laden bone models with biomimetic intrafibrillar collagen mineralization. Nat Commun. 2019;10 10.1038/s41467-019-11455-8 31388010 PMC6684598

[39] Henriques Lourenço A, Neves N, Ribeiro-Machado C, Sousa SR, Lamghari M, Barrias CC, et al Injectable hybrid system for strontium local delivery promotes bone regeneration in a rat critical-sized defect model. Sci Rep. 2017;7 10.1038/s41598-017-04866-4 28698571 PMC5506032

[40] Naddeo P, Laino L, La Noce M, Piattelli A, De Rosa A, Iezzi G, et al Surface biocompatibility of differently textured titanium implants with mesenchymal stem cells. Dent Mater. 2015;31: 235–243. 10.1016/j.dental.2014.12.015 25582059

[41] Matta C, Szűcs-Somogyi C, Kon E, Robinson D, Neufeld T, Altschuler N, et al Osteogenic differentiation of human bone marrow-derived mesenchymal stem cells is enhanced by an aragonite scaffold. Differentiation. 2019;107: 24–34. 10.1016/j.diff.2019.05.002 31152959

[42] de Oliveira Gonçalves J, Buchaim D, de Souza Bueno C, Pomini K, Barraviera B, Júnior R, et al Effects of low-level laser therapy on autogenous bone graft stabilized with a new heterologous fibrin sealant. J Photochem Photobiol B. 2016;162: 663–668. 10.1016/j.jphotobiol.2016.07.023 27497370

[43] Pomini KT, Andreo JC, De Rodrigues AC, De Gonçalves JBO, Daré LR, German IJS, et al Effect of low-intensity pulsed ultrasound on bone regeneration biochemical and radiologic analyses. J Ultrasound Med. 2014;33: 713–717. 10.7863/ultra.33.4.713 24658953

[44] Pomini KT, Buchaim DV, Andreo JC, Rosso MP de O, Della Coletta BB, German ÍJS, et al Fibrin Sealant Derived from Human Plasma as a Scaffold for Bone Grafts Associated with Photobiomodulation Therapy. Int J Mol Sci. 2019;20: 1761 10.3390/ijms20071761 30974743 PMC6479442

[45] Escudero JSB, Perez MGB, de Oliveira Rosso MP, Buchaim DV, Pomini KT, Campos LMG, et al Photobiomodulation therapy (PBMT) in bone repair: A systematic review. Injury. 2019;50: 1853–1867. 10.1016/j.injury.2019.09.031 31585673

