# Supplementary figures and images for: Elastin-derived scaffolding associated or not with bone morphogenetic protein (BMP) or hydroxyapatite (HA) in the repair process of metaphyseal bone defects
Source: PLoS One. 2020 Apr 20;15(4):e0231112. doi: 10.1371/journal.pone.0231112 (PMC7170266; doi:10.1371/journal.pone.0231112)

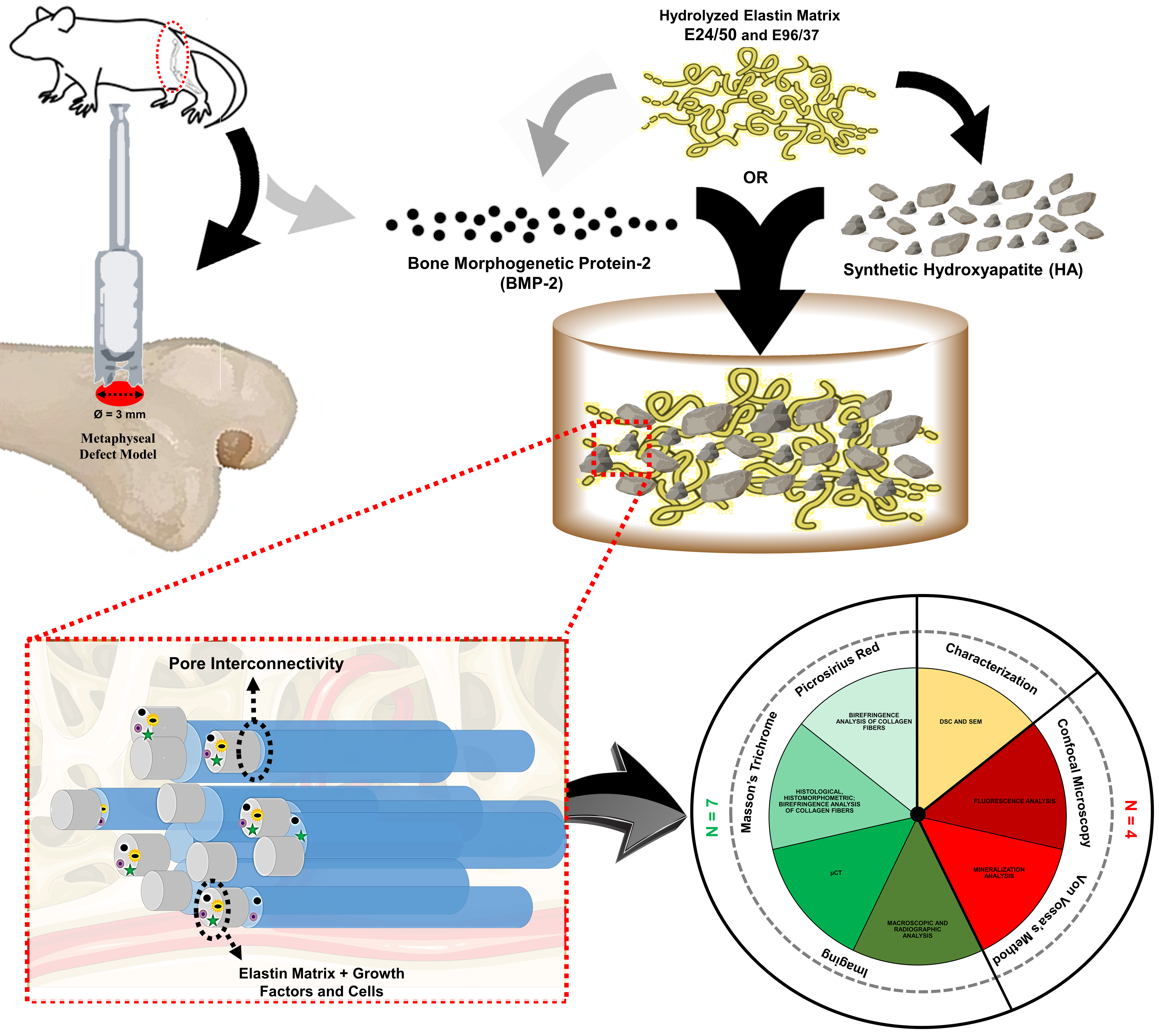

Supplement: S1 Graphical abstract — (TIF) [file pone.0231112.s001.tif]
